# Stromal micropapillary pattern predominant lung adenocarcinoma - a report of two cases

**DOI:** 10.1186/1746-1596-6-92

**Published:** 2011-09-29

**Authors:** Miki Ohe, Tomoyuki Yokose, Yuji Sakuma, Sachie Osanai, Chikako Hasegawa, Kota Washimi, Kimitoshi Nawa, Tetsukan Woo, Rurika Hamanaka, Haruhiko Nakayama, Yoichi Kameda, Kouzo Yamada, Takeshi Isobe

**Affiliations:** 1 Department of Pathology, Kanagawa Cancer Center, Kanagawa, Japan; 2 Department of Thoracic Oncology, Kanagawa Cancer Center, Kanagawa, Japan; 3 Molecular Pathology and Genetics Division, Kanagawa Cancer Center Research Institute, Kanagawa, Japan; 4 Division of Clinical Oncology and Respiratory Medicine, Department of Internal Medicine, Shimane University Faculty of Medicine, Shimane, Japan

**Keywords:** lung adenocarcinoma, micropapillary subtype, stromal micropapillary pattern, aerogeneous micropapillary pattern

## Abstract

Generally, adenocarcinomas with micropapillary pattern, featuring small papillary tufts lacking a central fibrovascular core, are thought to have poor prognosis. This pattern has been described in various organs. However, tumor cells with micropapillary pattern of lung adenocarcinoma are more often seen to float within alveolar spaces (aerogenous micropapillary pattern, AMP) than in fibrotic stroma like other organs (stromal micropapillary pattern, SMP) and SMP predominant lung adenocarcinoma (SMPPLA) has not been well described yet. We presented two cases of SMPPLA which were found in the last four years. Both the cases showed more than 50% of SMP in the tumor area. The majority of the stromal micropapillary clusters expressed MUC1 and epithelial membrane antigen along the outer surface of cell membrane. On the other hand, connective tissues surrounding stromal micropapillary clusters showed no reactivity for epithelial markers (thyroid transcription factor-1 and cytokeratin) or endothelial marker (D2-40 and CD34). It means clusters of SMP do not exist within air space or lymphatic or vessel lumens. The tumors with SMP often presented lymphatic permeation and vessel invasion, and intriguingly, one of the two cases showed metastasis to the mediastinal lymph node. Additionally, both the cases showed *EGFR *point mutations of exon 21. These results suggest that SMPPLA might be associated with poor prognosis and effective for EGFR tyrosine kinase inhibitors.

## Background

A new lung adenocarcinoma classification has been proposed by the International Association for the Study of Lung Cancer, American Thoracic Society and European Respiratory Society (IASLC/ATS/ERS). In this classification, the micropapillary subtype of lung adenocarcinoma (MSLA) was recommended as a newly added subtype of lung adenocarcinoma to lepidic, acinar, papillary, and solid subtypes defined in the 2004 World Health Organization (WHO) classification [1,2]. Generally, the micropapillary pattern is defined as tumor cells growing in papillary tufts, which lack fibrovascular cores surrounded by lacunar spaces and has been reported to be associated with a high incidence of nodal metastasis and poor prognosis [3-6]. This pattern has been described in various organs such as breast [7,8], urinary bladder [9,10], ovary [11,12], salivary gland [13], and is known to behave aggressively. In other organs than the lung, this pattern was observed mainly in stroma as invasive components (stromal micropapillary pattern: SMP) [7-19]; however in lung, MSLA is widely recognized as floating tumor cells within alveolar spaces (aerogenous micropapillary pattern: AMP) [3,4].

We examined whether SMP predominant subtypes were present in lung adenocarcinoma. During the period from February 2007 to December 2010, 559 patients with lung adenocarcinoma were consecutively treated by surgical resection at the Kanagawa Cancer Center, Kanagawa, Japan, and we found only two cases of SMP predominant lung adenocarcinoma (SMPPLA) (0.36%). We reported the cases of SMPPLA and attempted to describe the clinicopathological features.

## Case presentation

### Clinical summary

#### Case1

**Figure 1:**
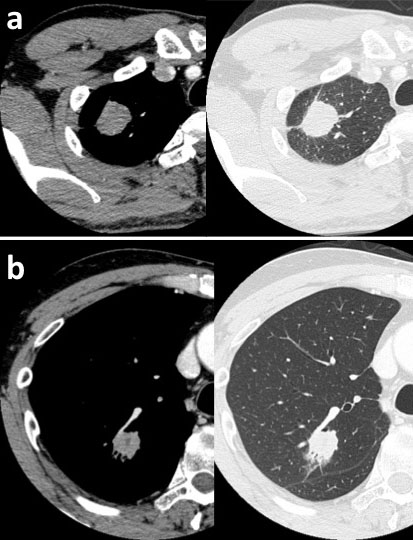
**Enhanced chest CT of the lung**. Chest CT of case 1 (a) and case 2 (b) showed a tumor of the right upper lobe of the lung. (a) Chest CT revealed a tumor with pleural indentation, without ground glass opacity (GGO). The tumor was 32 mm in diameter and mildly enhanced. (b) Chest CT revealed a nodule with GGO, pleural indentation, air bronchogram and venous involvement. The nodule was 29 mm in diameter and mildly enhanced.

A 49-year-old Japanese man was referred to the hospital with lung adenocarcinoma, which was diagnosed by the transthoracic needle biopsy. A computed tomography (CT) scan detected a 32 mm-sized localized solid tumor in the right upper lobe and swelling of the mediastinal lymph node (Figure 1a). He was an ex-smoker and admission laboratory tests showed increased carcinoembryonic antigen (9.6 ng/mL). A right upper lobectomy with lymph node dissection was performed and the tumor was diagnosed as lung adenocarcinoma in pathological T2aN2M0 and stage IIIA determined on the basis of the TNM classification of Union of International Cancer Control [20]. After that, he underwent postoperative adjuvant chemotherapy, and he was alive without recurrence ten months after operation.

#### Case2

A 57-year-old Japanese man who was a never smoker was referred to the hospital with abnormal shadow on his chest CT scan. A CT scan detected a 29 mm-sized localized solid nodule with pleural indentation in the right upper lobe, and the histological diagnosis of the tumor by transbronchoscopic biopsy was lung adenocarcinoma (Figure 1b). Laboratory tests showed slightly elevated squamous cell carcinoma antigen (1.6 ng/mL). A right upper lobectomy with lymph node dissection was performed and diagnosed lung adenocarcinoma in pathological T2aN0M0 and stage IB. After that, he underwent postoperative adjuvant chemotherapy, and he was alive without recurrence nine months after operation.

### Pathological findings

The excised specimens were fixed in a solution of 10% buffered formaldehyde and the sections were embedded in paraffin. Four micrometer-thick sections including the largest cut surface of the tumor were prepared and stained with hematoxylin and eosin (HE), alcian blue and elastica-van-Gieson (AB-EVG) stain. Immunohistochemical staining was performed with the primary antibodies listed in Table 1. Lepidic, acinar, papillary and solid subtypes of lung adenocarcinoma were determined according to the 2004 WHO classification. AMP of lung adenocarcinoma was determined according to the IASLC/ATS/ERS international multidisciplinary classification of lung adenocarcinoma [1].

**Table 1:** Antibodies used and immunohistchemical result.


**Antibody**	**Clone**	**Dilution**	**Source**	**Stroma around the micropapillary clusters**		**Tumor cells**			
						**in SMP**		**outside SMP**	
				
				**Case1**	**Case2**	**Case1**	**Case2**	**Case1**	**Case2**

MUC1	Ma695	1:100	Novocastra	0	0	2+	2+	1+	2+
EMA	E29	Pre-diluted	Cell Marque	0	0	2+	2+	2+	2+
E-cadherin	NCH38D	1:100	DakoCytomation	0	0	2+	2+	2+	2+
CK	AE1/AE3	Pre-diluted	Nichirei	0	0	2+	2+	2+	2+
TTF-1	8G7G3/1	1:100	DakoCytomation	0	0	1+	2+	2+	2+
SP-A	PE10	1:100	Dako	0	0	1+	0	2+	2+
D2-40	D2-40	Pre-diluted	Nichirei	0	0	0	0	0	0
CD34	Nu-4A1	Pre-diluted	Nichirei	0	0	0	0	0	0
FactorVIII	polyclonal	1:200	DAKO	0	0	0	0	0	0

EMA, epithelial membrane antigen; CK, cytokeratin; TTF-1, thyroid transcription factor-1; SP-A, surfactant protein A.

Novocastra, Newcastle Upon Tyne, UK; Cell Marque, Hot springs, AR, USA; DakoCytomation, Carpinteria, CA, USA; Dako, Kyoto, Japan; Nichirei, Tokyo, Japan; DAKO, Glostrup, Denmark.

0, no positive cells; 1+, positive tumor cells less than 10%; 2+, 10% or more.

**Figure 2:**
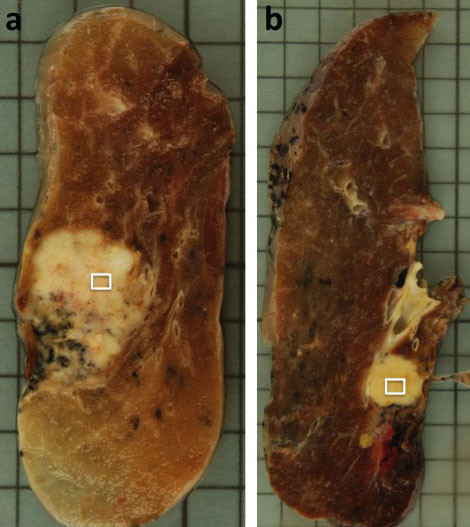
**Macroscopic findings**. Macroscopic examination of excised specimens showed the tumor measuring 36 × 25 × 30 mm in size, white in color on cut surface in the case 1 (a), and the tumor measuring 33 × 20 × 40 mm in size, grayish-white in color on cut surface containing areas of hemorrhage in the case 2 (b). White squares in Figure 2a and 2b correspond to photomicrographs of Figure 3a and 3b, respectively.

Macroscopic examination of the excised specimens showed the sharply-demarcated tumor measuring 36 × 25 × 30 mm in size, white in color on cut surface in the case 1 (Figure 2a), and the tumor measuring 33 × 20 × 40 mm in size, grayish white in color on cut surface in the case 2 (Figure 2b).

**Figure 3:**
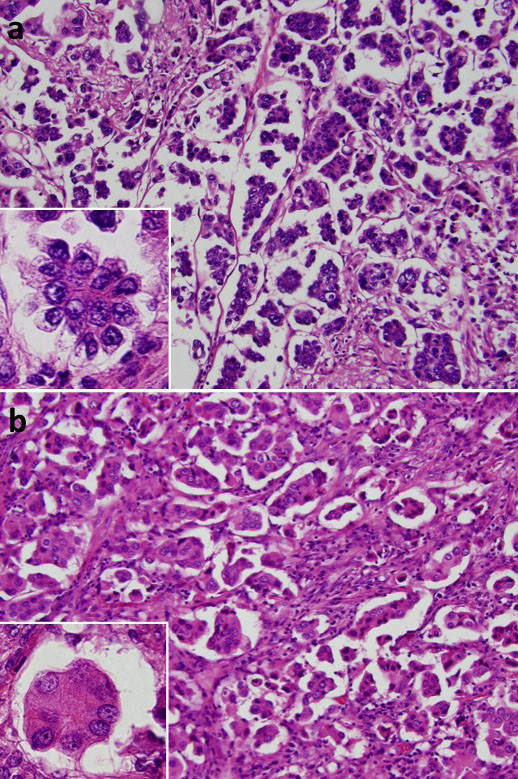
**Photomicrographs**. Stromal micropapillary pattern characterized by papillary structures with tufts lacking a central fibrovascular core and surrounding clear spaces in fibrotic stroma was seen in both case 1 (a) and case 2 (b). (HE stains ×100; inset, ×400)

**Figure 4:**
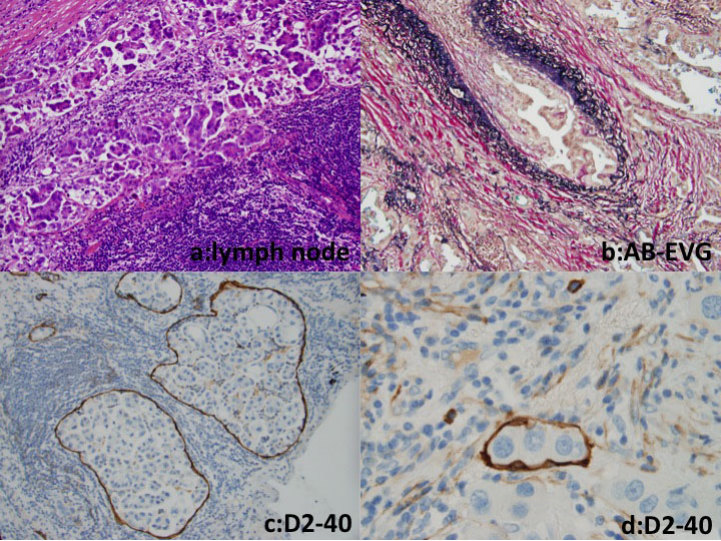
**Photomicrographs**. (a) A resected regional lymph node had metastatic foci composed of tumor cells with a micropapillary pattern in the case 1. (HE stain, ×200) (b) Vessel invasion by tumor cells in the case 1. (AB-EVG stain, ×100) (c, d) Tumor cells in lymphatic ducts, which were covered by D2-40 positive endothelial cells. (c, ×200, case 1; d, ×400, case 2)

Microscopically, the tumors of both the cases were composed of stromal micropapillary predominant adenocarcinoma, which were proliferation of nonmucinous atypical cuboidal epithelial cells. In the case 1, the tumor was composed of 55% SMP, 5% lepidic, 30% acinar, 5% papillary pattern and 5% AMP (Figure 3a). In the case 2, the tumor was composed of 55% SMP, 20% lepidic and 25% acinar pattern (Figure 3b) and lacked AMP. In both the cases, lymphatic and vessel invasion were often observed. Lymphatic invasion was confirmed on immunohistochemistry using D2-40 antibody. The case 1 showed pleural invasion and resected regional lymph nodes had metastatic foci composed of tumor cells with a micropapillary pattern (Figure 4).

**Figure 5:**
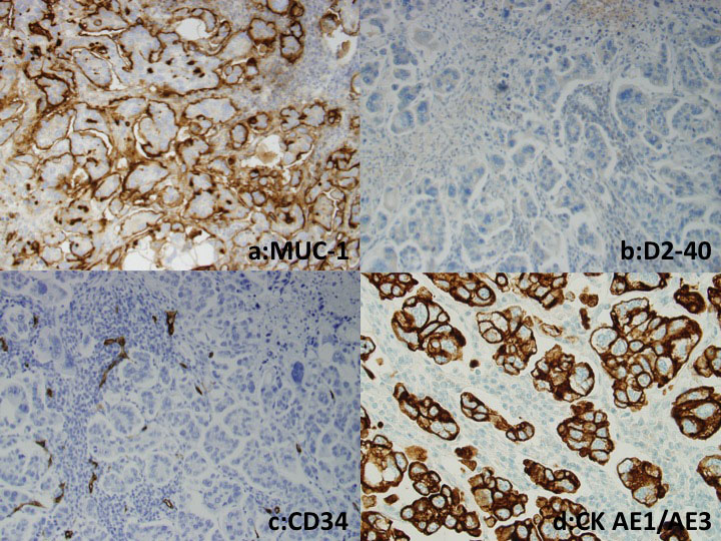
**Immunohistochemical photomicrographs of case 1**. (a) Tumor clusters of stromal micropapillary pattern had MUC1 expression on the outer surface strongly, indicating 'inside-out' pattern. (×200) (b) D2-40 and (c) CD34 were not found in cells consisting micropapillary tufts and (d) CK was not found in connective tissue surrounding the tumor cells. (b, c, ×200; d, ×400)

Immunohistochemically, the outer surface of the SMP cell clusters in both the cases showed membranous expression of MUC1 and epithelial membrane antigen (EMA), indicating an 'inside-out' pattern. Both the cases showed E-cadherin expression on intercellular cell membranes of micropapillary tufts of SMP tumor cells. Tumor cells constituting SMP showed positive staining for Ki-67 (MIB-1, Dako, Glostrup, Denmark); positive rate were 24% in case 1, 62% in case 2. In SMP component, positive and negative tumor cells for thyroid transcription factor-1 (TTF-1) and surfactant protein A (SP-A) staining were observed in the case 1, on the other hand, the case 2 showed TTF-1 expression and SP-A repression in almost all tumor cells. Tumor cells outside SMP showed MUC1 and EMA expression on free surface of cell membrane, but MUC1 positive cells were fewer than those in SMP. Tumor cells outside SMP in case 1 showed strong TTF-1 expression. D2-40, CD34 and factor VIII were not found in cells constituting micropapillary tufts. Moreover TTF-1, cytokeratin (CK), D2-40, CD34 and factor VIII were negative in luminal inner surface surrounding micropapillary tufts (Table 1 Figure 5a-d). These results means micropapillary tufts of SMP lack fibrovascular core and were not located within alveolar space, vascular or lymphatic vessels.

### Mutational analysis

A study of epidermal growth factor receptor (*EGFR*) gene status of exon 19 and 21 was performed by the methods described elsewhere [21]. Written informed consent for genetic analysis of the tumor cells was obtained from the patients. As a result, both the cases showed an L858R point mutation at exon 21.

## Discussion

In the two cases presented, we recognized that tumor cells of SMP had reduced expression of SP-A, but tumor cells outside SMP had its strong expression. Additionally, we identified the tumor cluster cells were surrounded by the connective tissue which was negative for TTF-1 or CK. And these components were also negative for D2-40 and CD34. The results suggested micropapillary tufts of SMP were not located within alveolar space, vascular or lymphatic vessels but in stroma and reduced the phenotypic expression like SP-A [22].

AMP of MSLA has been reported to express of MUC1 [6]. We identified expression of MUC1 and EMA in the outer surface of papillary clusters like those in AMP of the case 1. This staining pattern is called as an 'inside-out' pattern in the invasive micropapillary carcinoma of breast [7,8] and several organs [9-19]. Therefore, the polarity called as an 'inside-out' pattern of the tumor cells is thought to be a characteristic feature in SMP and AMP of lung adenocarcinoma.

Previous studies have reported that a micropapillary pattern is associated with a poor prognosis in stage I lung adenocarcinoma because of its aggressive behavior, as shown by frequent lymph node metastasis, lymphatic permeation, vascular invasion and pleural invasion [4-6]. In the current two cases, lymphatic permeation and vascular invasion were often observed. In addition, the case 1 showed pleural invasion and the resected regional lymph node had metastatic foci composed of tumor cells with a micropapillary pattern. Though it is too early to refer to prognosis of SMPPLA because of short duration of observation, we may say SMPPLA has strong association with vascular invasion.

We also performed a mutational analysis of *EGFR *gene mutations and both the cases showed *EGFR *mutations of exon 21. These results suggest that SMPPLA might be associated with poor prognosis and effective for EGFR tyrosine kinase inhibitors.

In conclusion, we recognized the presence of SMPPLA. Since SMPPLA is very rare as far as we investigated, further studies are required to determine the clinical significance of SMPPLA in detail.

## Consent

Written informed consent was obtained from the patients for publication of this case report and any accompanying images. A copy of the written consent is available for review by the Editor-in-Chief of this journal.

## Abbreviations

AMP: aerogenous micropapillary pattern; SMP: stromal micropapillary pattern; SMPPLA: SMP predominant lung adenocarcinoma; MSLA: the micropapillary subtype of lung adenocarcinoma

## Competing interests

The authors declare that they have no competing interests.

## Authors' contributions

MO and TY designed the study, performed clinical and pathological investigation, and wrote the drafts. YS participated in pathological and genetical investigation. SO performed the histological and immunohistochemical evaluation. CH, KW, KN, TW and RH assisted the clinical investigation. HN participated in managing and operating the patients. YK assisted the pathological investigation. KY participated in collecting clinical data and images. TI participated in its design and coordination and helped to draft the manuscript. All authors read and approved the final manuscript.
